# Macrodiolide Formation by the Thioesterase of a Modular Polyketide Synthase**

**DOI:** 10.1002/anie.201500401

**Published:** 2015-03-06

**Authors:** Yongjun Zhou, Patrícia Prediger, Luiz Carlos Dias, Annabel C Murphy, Peter F Leadlay

**Affiliations:** Department of Biochemistry, University of Cambridge80 Tennis Court Road, Cambridge CB2 1GA (UK); Institute of Chemistry, State University of Campinas, UNICAMPC.P. 6154, CEP 13084-971, Campinas SP (Brazil)

**Keywords:** biosynthesis, diolides, elaiophylin, polyketide synthase, thioesterase

## Abstract

Elaiophylin is an unusual C_2_-symmetric antibiotic macrodiolide produced on a bacterial modular polyketide synthase assembly line. To probe the mechanism and selectivity of diolide formation, we sought to reconstitute ring formation in vitro by using a non-natural substrate. Incubation of recombinant elaiophylin thioesterase/cyclase with a synthetic pentaketide analogue of the presumed monomeric polyketide precursor of elaiophylin, specifically its *N*-acetylcysteamine thioester, produced a novel 16-membered C_2_-symmetric macrodiolide. A linear dimeric thioester is an intermediate in ring formation, which indicates iterative use of the thioesterase active site in ligation and subsequent cyclization. Furthermore, the elaiophylin thioesterase acts on a mixture of pentaketide and tetraketide thioesters to give both the symmetric decaketide diolide and the novel asymmetric hybrid nonaketide diolide. Such thioesterases have potential as tools for the in vitro construction of novel diolides.

Modular type I polyketide synthases (PKSs) are giant multifunctional enzymes, principally from actinomycete bacteria, that use a remarkable assembly-line logic for the biosynthesis of a diverse array of bioactive natural products,[1] including a number of clinically valuable antibiotics, immunosuppressants, and anticancer compounds. Each module contains a ketosynthase (KS), which condenses activated acyl and malonyl units; an acyltransferase (AT), which specifies the type of extender unit introduced; and an acylcarrier protein (ACP), which tethers the growing polyketide chain while it is processed by optional ketoreductase (KR), dehydratase (DH), and enoylreductase (ER) domains. The processed intermediates are passed from module to module until the full-length linear chain is released, most commonly through the action of a thioesterase/cyclase (TE) domain.[2]

The directness of the link between the PKS gene sequence and the chemical structure of the end product has revolutionized our view of the evolution of antibiotic biosynthesis,[3] and has stimulated ongoing efforts to expand polyketide structural diversity by reprogramming modular assembly lines.[4] It is particularly important to understand the specificity of chain-terminating TE domains, since these enzymes have a controlling influence on whether reprogrammed polyketide products are efficiently released, and on whether cyclization is favored over hydrolysis.[5] Previous in vitro work has been carried out on the TE domain that catalyzes formation of the siderophore enterobactin[6] and on the assembly-line TE domains for several nonribosomal peptide synthetases[7] and the results show that such enzymes have a fairly relaxed specificity and can be deployed as cyclization catalysts. The X-ray crystal structures have been determined for chain-terminating TE domains from the PKS assembly lines for both macrocyclic polyketides[8] and linear polyketides,[9] thus providing a valuable framework for mechanistic investigation. The ability of several individual polyketide TE domains to catalyze the in vitro macrocyclization of thioester substrates has also been demonstrated.[5a,c, 10] However, we are still far from a detailed understanding of the factors that influence specificity and selectivity for these enzymes.

An intriguing and relatively rare variation in the mode of polyketide release from modular PKS assembly lines leads to *C*_2_-symmetric macrocyclic dilactones, or diolides.[11] *C*_2_-symmetric diolides of diverse ring size have now been characterized from numerous sources including bacteria,[12] fungi,[13] and marine animals (or their commensal microorganisms).[14] A better understanding of the molecular basis for such catalysis might enable a novel mild chemoenzymatic route to non-natural analogues of such compounds. We report herein the cloning, expression, and in vitro dimerizing activity of the chain-terminating TE domain of the modular PKS multienzyme that synthesizes the 16-membered diolide (**1 b**; Scheme 1) of elaiophylin,[2c, 15] a compound with antibacterial, antiviral, antifungal, and immunomodulatory activities.

**Scheme 1:**
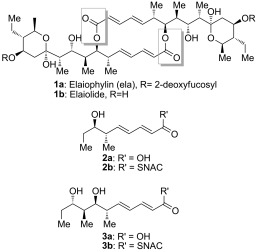
The structures of elaiophylin 1 a, elaiolide 1 b, and the analogues of the monomeric polyketide precursor of elaiolide: tetraketide 2 and pentaketide 3.

Two alternative mechanisms can be advanced for formation of the symmetrical diolide aglycone **1 b** on the polyketide synthase, as illustrated in Scheme 2. In route 1), initial nucleophilic attack by the distal hydroxy group of the TE-bound monomer on the ACP-bound thioester affords the linear dimer attached to the TE active site ready for cyclization. In route 2), the TE-bound monomer is attacked by the distal hydroxy group of the ACP-bound monomer to give the linear dimer attached to the ACP (“retrotransfer”), and then the linear dimer is transferred to the vacant TE active site for cyclization. This retrotransfer or iterative mechanism (route 2)) has previously been demonstrated for the cyclization steps of nonribosomal peptide synthetases.[7b,c] By using *N*-acetylcysteaminyl thioesters (SNAC thioesters) of tetraketide and pentaketide analogues of the natural octaketide monomers, which in vivo are acted upon by the TE while tethered to an adjacent ACP domain in the multienzyme assembly line, we show here that the TE can catalyze homodimerization of the synthetic pentaketide **3 b** (Scheme 1) to a novel 16-membered decaketide diolide **5** (Figure 1), and we identify an intermediate that sheds light on the enzymatic mechanism. Although the tetraketide thioester **2 b** (Scheme 1) is not itself a substrate for homodimerization, the substrate flexibility of the elaiophylin TE (Ela-TE) is further shown by the fact that in the presence of both **2 b** and **3 b**, a novel asymmetric nonaketide **6** is formed in addition to the expected decaketide **5** (Figure 2).

**Figure 1:**
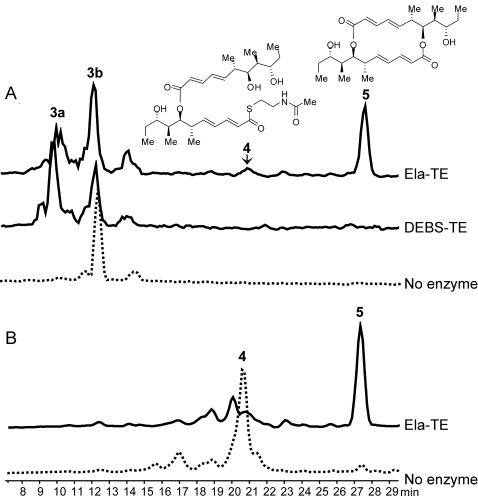
HPLC–MS analysis of the products of Ela-TE action on model substrates. A) Compounds 4 and 5, as well as the hydrolysis product 3 a, are generated from 3 b by Ela-TE. DEBS-TE exclusively catalyzes hydrolysis to 3 a. B) Compound 4, when purified from the reaction mixture and re-incubated with fresh Ela-TE, is cyclized into 5.

**Figure 2:**
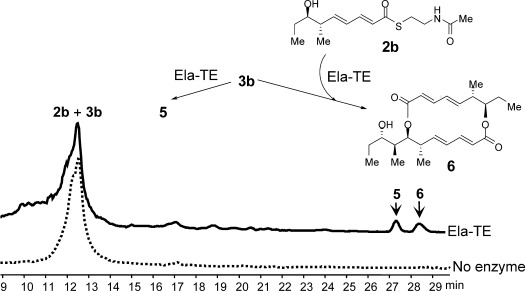
HPLC–MS analysis of the products of Ela-TE action on an equimolar mixture of 2 b and 3 b. The novel asymmetric 16-membered macrodiolide nonaketide 6 was produced from 2 b and 3 b and 5 was generated from 2 b alone.

**Scheme 2:**
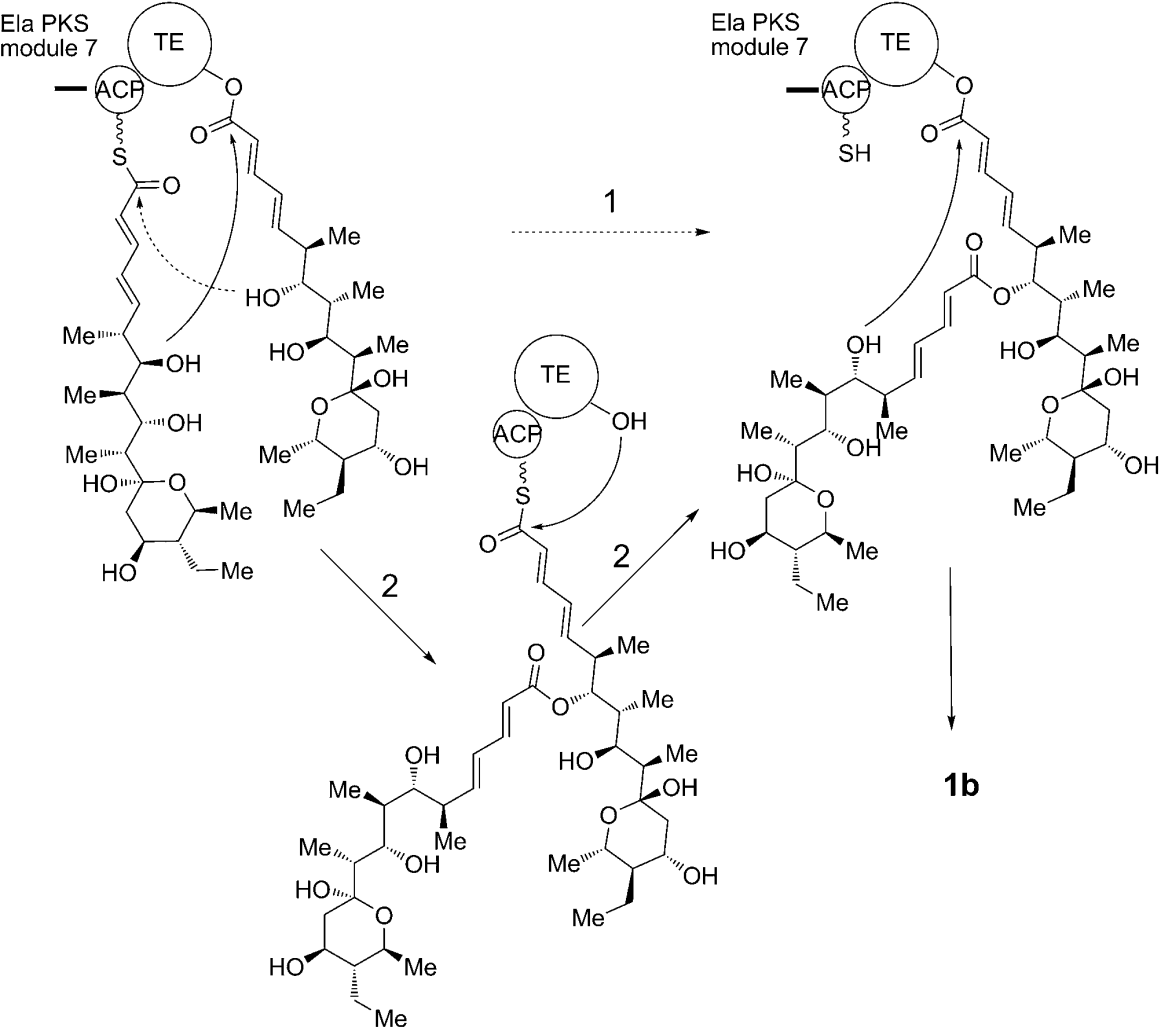
Alternative mechanisms for the formation of symmetrical diolide 1 b. Route 1): initial nucleophilic attack by the distal hydroxyl group of the TE-bound monomer on the ACP-bound thioester. Route 2): the TE-bound monomer is attacked by the distal hydroxy group of a second monomer tethered to the adjacent ACP domain.

Candidate substrates for the diolide cyclase were obtained through stereoselective synthesis of analogues of the elaiophylin monomeric seco acid (Scheme 1). Tetraketide SNAC thioester **2 b** was obtained in 12 steps (overall yield 18.3 %), while pentaketide SNAC thioester **3 b** was obtained in 13 steps (overall yield 8.0 %; Scheme 3 and Supporting Information, Section 3. The chain-terminating TE domain from the previously-characterized elaiophylin PKS[15] was obtained from *E. coli* as a soluble protein of the expected molecular mass (Figure S1 in the Supporting Information). In contrast to previously studied TE domains from macrocyclic PKS multienzymes, which retain a dimeric structure,[8] the elaiophylin TE was found to be largely monomeric in solution (Figure S2).

**Scheme 3:**
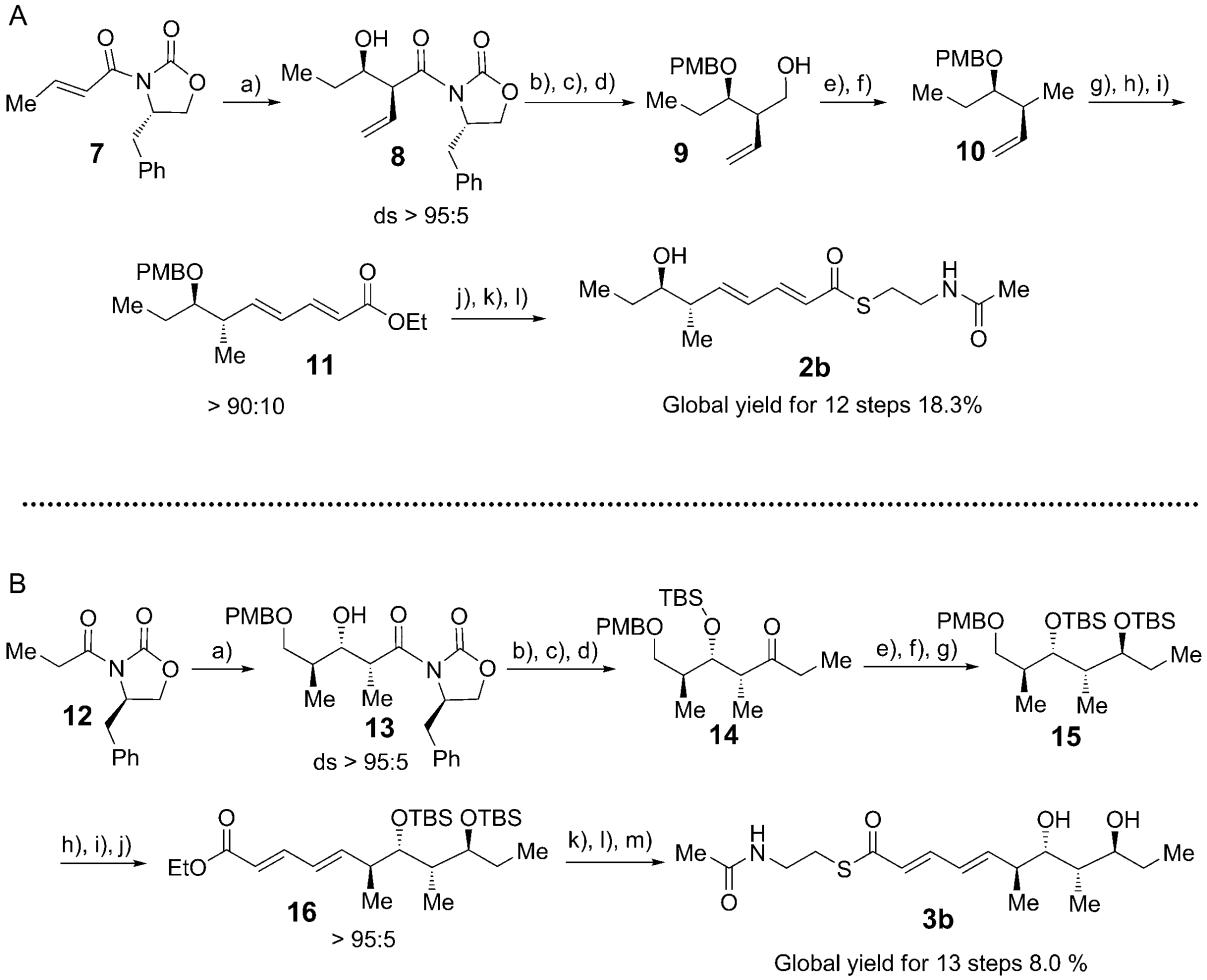
Synthesis of tetraketide 2 b and pentaketide 3 b as model substrates for Ela-TE. Reagents and conditions: A) a) i. *n*Bu_2_BOTf, TEA, CH_2_Cl_2_, −78 °C, ii. EtCHO, −78 °C to 0 °C, 3 h, 65–68 % (ds>95:5). b) LiBH_4_, THF, MeOH, 45 mins, 0 °C. c) 1-(dimethoxymethyl)-4-methoxybenzene, CSA, CH_2_Cl_2_, RT, 93 % (for two steps). d) DIBAL, CH_2_Cl_2_, 45 mins, 0 °C, 99 %. e) TsCl, DMAP, TEA, CH_2_Cl_2_, RT, 4 h, 90 %. f) LiBH_4_, THF, 0 °C to RT, 24 h, 85 %. g) OsO_4_, NMO, buffer pH 7, THF/acetone, 5 h, RT. h) NaIO_4_, buffer pH 7, THF, 12 h, (86 % for two steps). i) i. LiHMDS, THF, −78 °C to −25 °C, 30 mins, ii. (*E*)-ethyl 4-(diethoxyphosphoryl)but-2-enoate, THF, −78 °C to −25 °C, 84 %. j) KOH, EtOH/H_2_O, 12 h, RT, 99 %. k) *N*-(2-*m*ercaptoethyl)acetamide, DCC, HOBt, DMF, 0 °C to RT, 12 h, 67 %. l) DDQ, buffer pH 7, DCM, 0 °C, 80 %. B) a) i. *n*Bu_2_BOTf, DIPEA, CH_2_Cl_2_, −10 °C, ii. −78 °C, (*S*)-3-((4-methoxybenzyl)oxy)-2-methylpropanal, 70 %(ds>95:5). b) *N*,*O*-dimethylhydroxylamine hydrochloride, Me_3_Al, THF, 6 h, 0 °C to RT, 72 %. c) TBSOTf, CH_2_Cl_2_, 2,6-lutidine, 1 h, 0 °C to RT, 79 %. d) EtMgBr, THF, 5 h, 0 °C, 78 %. e) HF⋅Py, THF, 0 °C to RT, 12 h, 95 %. f) Me_4_NBH(OAc)_3_, MeCN, AcOH, 12 h, −30 °C to −20 °C, 88 %. g) TBSOTf, 2,6-lutidine, CH_2_Cl_2_, 1 h, 0 °C to RT, 87 %. h) DDQ, buffer pH 7, CH_2_Cl_2_, 0 °C, 82 %. i) (COCl)_2_, DMSO, TEA, CH_2_Cl_2_, −78 °C, 2 h, 86 %. j) i. LiHMDS, THF, −78 °C to −25 °C, 30 mins, ii. (*E*)-ethyl 4-(diethoxyphosphoryl)but-2-enoate, THF, −78 °C to −25 °C, 2 h, 94 %. k) KOH, EtOH/H_2_O_,_ 12 h, RT, 99 %. l) *N*-(2-*m*ercaptoethyl)acetamide, DCC, HOBt, DMF, 0 °C to RT, 12 h. m) HF⋅Py, THF, 0 °C to RT, 12 h, 51 % (two steps). ds=diastereoselectivity. TEA=triethylamine, CSA=10-camphorsulfonic acid, DIBAL=diisobutylaluminum hydride, Ts=4-toluenesulfonyl, DMAP=4-dimethylaminopyridine, NMO=*N*-methylmorpholine-*N*-oxide, DCC=1,3-dicyclohexylcarbodiimide, HOBt=1-hydroxybenzotriazole, DMF=*N*,*N*-dimethylformamide, DDQ=2,3-dichloro-5,6-dicyano-1,4-benzoquinone, DIPEA=*N*,*N*-diisopropylethylamine, TBSOTf=*tert*-butyldimethylsilyl trifluoromethanesulfonate, LiHMDS=lithium hexamethyldisilazane.

Incubation of **3 b** (3 mm) with Ela-TE (40 μm) in 0.1 m potassium phosphate buffer (pH 8.2) containing 10 % DMSO produced, in a time- and enzyme-dependent manner, the symmetric decaketide diolide **5**, the structure of which was confirmed by HRMS and 1D- and 2D-NMR techniques (Supporting Information, Section 4.2); as well as the hydrolysis product **3 a**. These were accompanied by a further species eluted after 20.5 min (Figure 1 A), the concentration of which initially rose and then levelled off during the incubation (Figure S3). The structure of this species, as determined by HRMS and 1D- and 2D-NMR, corresponded to the linear dimer **4** (Supporting Information, Section 4.1). Notably, dimerization of **3 b** gave only the symmetrical 16-membered macrodiolide, as found in natural **1 b**, with no evidence of regioisomers with a different ring size being formed. We also tested the chain-terminating cyclase/thioesterase from the erythromycin pathway (DEBS-TE)[16] as a potential catalyst for the dimerization of **3 b**, but this enzyme exclusively catalyzed hydrolysis to **3 a** (Figure 1 A).

When **4** was purified from the reaction mixture and re-incubated with fresh Ela-TE, it was cyclized into **5** (Figure 1 B), a result consistent with **4** being an essential intermediate in the macrocyclization of **3 b**. As a control, we separately determined that purified **5** was stable to hydrolysis by Ela-TE under these experimental conditions (data not shown). These observations show that the TE is competent to catalyze both the ligation of two monomeric polyketide chains and subsequent diolide formation and they support the mechanism of route 2) (Scheme 2) for macrodiolide formation in vitro since route 1) would not generate **4**. We propose that the same iterative mechanism operates in vivo, especially since the published structures for dimeric PKS TE domains[8] reveal that functional communication between the TE active sites is highly improbable.

In contrast to pentaketide analogue **3 b**, the tetraketide **2 b** yielded only the hydrolysis product **2 a** upon incubation with Ela-TE (data not shown). Interestingly, when an equimolar mixture of **2 b** and **3 b** was incubated with Ela-TE under the same conditions, the asymmetric 16-membered macrodiolide nonaketide **6**, the structure of which was confirmed by HRMS and 1D- and 2D-NMR (Supporting Information, Section 4.3), was produced in addition to **5** and in comparable amounts (Figure 2).

To the best of our knowledge, this is the first example of a “hybrid” macrodiolide polyketide produced enzymatically in vitro. In this experiment, LC–MS revealed the presence of a species with the mass predicted for a linear nonaketide thioester with the same retention time as **4** (Figure S4). However insufficient material was available to allow NMR analysis. Further work will thus be required to determine the exact course of the reaction.

Previous structural studies on PKS TE domains catalyzing either hydrolysis[9] or macrocyclization[8] have identified the active site as lying within an unusual channel that traverses the entire protein. There are subtle differences in the size and shape of this channel in different structures but it remains difficult to identify individual enzyme–substrate interactions that determine the outcome. The Ela-TE domain shares the secondary structure and conserved sequence motifs of PKS TE domains of known 3D structure[8,9] that catalyze hydrolysis or macrocyclization (Figure S5). Furthermore, threading of the Ela-TE sequence onto these experimentally determined structures by using Phyre2[17] predicts with confidence that this TE likewise has the hallmark active-site channel (Figure S6). Consistent with this, a recent phylogenetic analysis of 138 TE domains for PKS and NRPS assembly-line systems showed that TE domains do not cluster based on substrate specificity or function, thus hinting at a model of convergent evolution towards (for example) macrodiolide formation.[18] These authors reported that when presented with a non-natural seco-acid, DEBS1-TE produces not only the 14-membered macrolactone and the hydrolysis product as major products, but also minor amounts of linear dimer and a 28-membered macrodiolide,[18] an observation fully in accord with our present results.

The iterative Ela-TE must catalyze a total of two acylation and two deacylation reactions to form the diolide, and further structural and functional studies will be needed to understand the respective selectivity of these steps. Nevertheless, our present results open the perspective of using such diolide TE domains preparatively to synthesize not only novel macrodiolides but also linear dimeric or even trimeric polyketide esters. Such studies with the purified TE domain of the oxazole diolide conglobatin[19] are underway in this laboratory and the results will be reported in due course.
